# Intramedullary Spinal Cord Abscess in an Adult Patient: A Case Report

**DOI:** 10.7759/cureus.73418

**Published:** 2024-11-11

**Authors:** Alexandros Moniakis, Christos Zlatanos, Giorgos Georgoulis

**Affiliations:** 1 Department of Neurosurgery, Athens General Hospital ‘Georgios Gennimatas’, Athens, GRC

**Keywords:** abscess, intramedullary, paraparesis, rehabilitation, spinal cord

## Abstract

Intramedullary abscess of the spinal cord (IASC) is a rare but treatable condition that presents with a variety of symptoms. An MRI scan with contrast is the imaging examination of choice. We present a rare case of IASC causing severe paraparesis in an otherwise healthy young male. As highlighted by this case report, prompt antimicrobial therapy and surgical evacuation can lead to favorable outcomes. Since IASC remains an entity poorly understood, reporting more similar cases should lead to the creation of a larger database that could deduce significant conclusions regarding its comprehension and treatment.

## Introduction

Intramedullary abscess of the spinal cord (IASC) is a rare infection of the central nervous system (CNS), first described in 1830 [1]. Although antibiotic use, radiographic imaging, and prompt surgical intervention have reduced mortality and morbidity significantly in comparison to the pre-antibiotic era [2], it still remains a condition that can cause a significant neurologic deficit [3]. The majority of the reported cases in the literature describe an acute onset of neurological deterioration depending on the location of the IASC, while the most common way of transmission is either hematogenous or contiguous [2,3]. MRI with contrast is the radiographical imaging of choice, and on most occasions, a ring-enhancing intramedullary lesion can be observed. Diffusion-weighted imaging (DWI) is a rather useful sequence in the diagnosis of this condition since it can reveal diffusion restriction, consistent with the infectious cause of the lesion. The most common pathogens identified were Mycobacterium tuberculosis, Streptococcus sp., and Staphylococcus sp. [3,4]. The treatment options offered to these patients are mainly antimicrobial therapy, with the assistance of surgical evacuation of the lesion when necessary [3,4]. This report discusses a case of this rare entity (IASC) in an otherwise healthy young male with a history of relapsed otitis. We aim to provide insights into its clinical presentation and management strategies, highlighting the favorable outcome following prompt treatment.

## Case presentation

**Figure 1:**
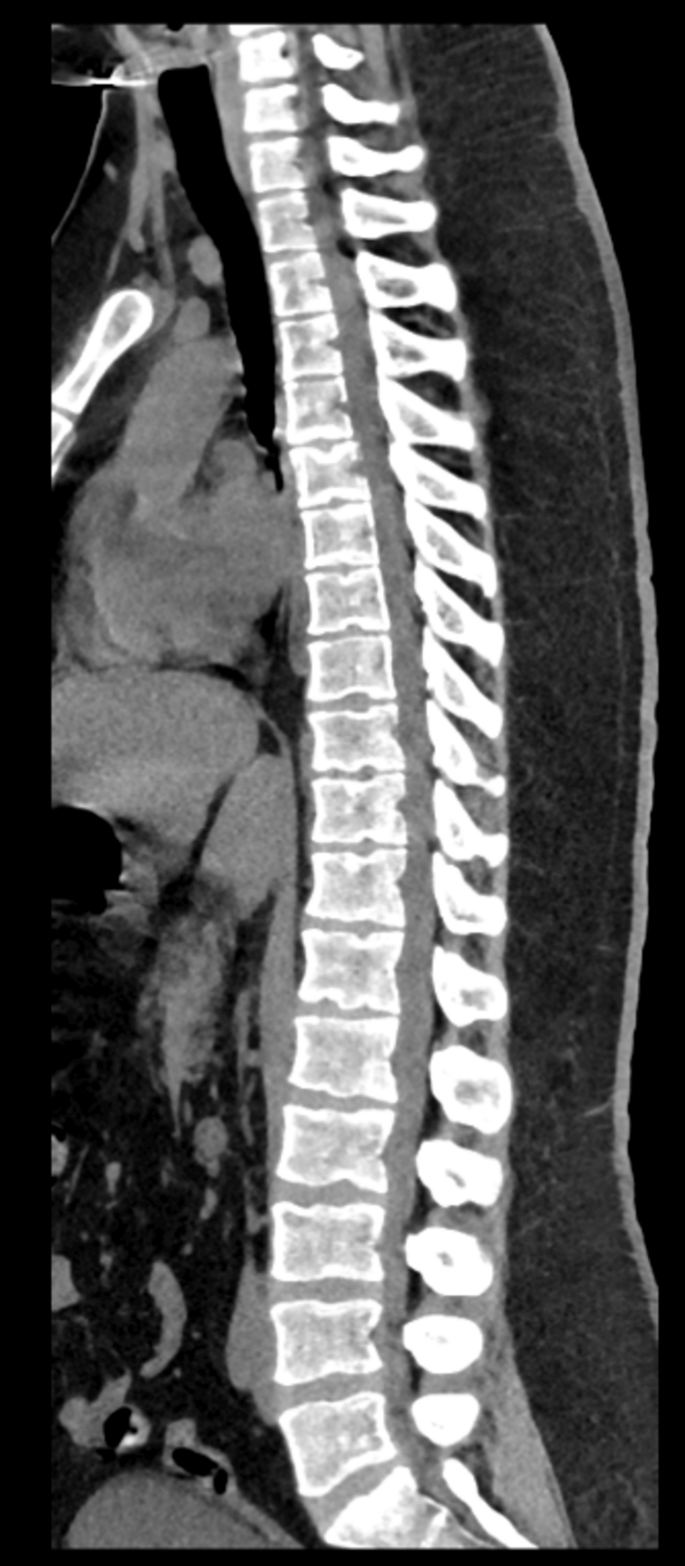
CT scans revealing no abnormal findings from the imaging of the spine.

A 20-year-old patient with a history of right otitis that required surgical evacuation three years ago was admitted to our department due to acute paraparesis combined with deteriorating malaise, which started twenty days before admission. His recent medical history mentions an episode of relapsed left otitis, which received antibiotic treatment two months prior to his admission. It should be noted that this infection was complicated by left Bell’s palsy, which resolved completely after steroid administration. On examination, he was febrile, hemodynamically stable, and with severe paraparesis (Medical Research Council (MRC) muscle power 3/5 in proximal muscle groups, 1/5 in distal muscle groups on the left lower extremity, and 1/5 in proximal muscle groups, 3/5 in distal muscle groups on the right lower extremity), as can be seen in Table 1. Moreover, his inflammatory markers were significantly elevated (WBC: 14.00 K/μL, normal values: 4.00-11.00 K/μL; and C-reactive protein: 170 mg/L, normal values: 0-6 mg/L). The initial CT scans performed in the emergency department revealed only diffuse opacification of the left mastoid cells and no abnormal findings from the imaging of the spine (Figure 1).

**Table 1:** Lab results of the patient highlighting the elevated inflammatory markers.

Laboratory parameter	Patient value	Normal value
Hemoglobin (Hb) g/dl	14.5	13.5-17.5
Hematocrit (Ht) %	44.7	41.0-53.0
White blood cells (WBCs) K/μl	14.00	4.00-11.00
Platelets (PLTs) K/μl	203	150-400
Urea mg/dl	38	16.6-48.5
Creatinine mg/dl	0.85	0.7-1.2
Sodium (Na) mmol/l	141	136-146
Potassium (K) mmol/l	4.7	3.5-5.1
C-reactive protein (CRP) mg/l	170	<3.8
Lactate dehydrogenase (LDH) U/l	147	135-225
Creatine phosphokinase (CPK) U/l	221	39-308

**Figure 2:**
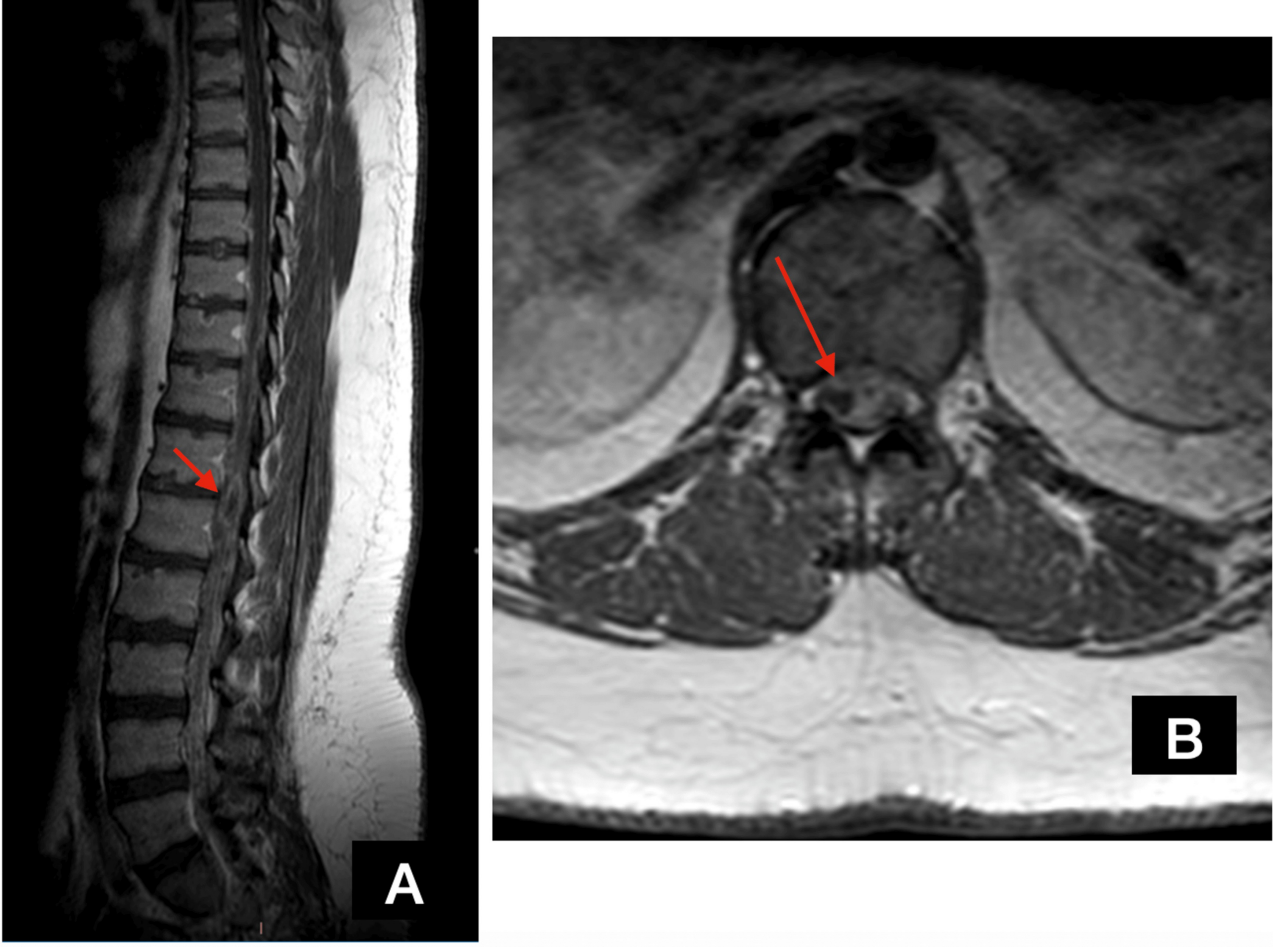
MRI scan findings. A: sagittal post-contrast T1 MRI scan showing a well-defined ring-enhancing lesion at the level of T12-L1; B: axial post-contrast T1 MRI scan of the same lesion

The patient was started on blind antimicrobial treatment with ceftriaxone. Unfortunately, on the second day of his hospitalization, a severe deterioration of his paraparesis was noted (MRC muscle power 1/5 in proximal muscle groups and 0/5 in distal muscle groups in both lower limbs) with concomitant hypoesthesia below the T12 level. An urgent MRI scan was performed, which revealed an intramedullary lesion (25 x 6 x 4 mm) at the T12-L1 level, with ring enhancement after IV gadolinium administration and excessive edema in the conus medullaris area (Figure 2).

**Figure 3:**
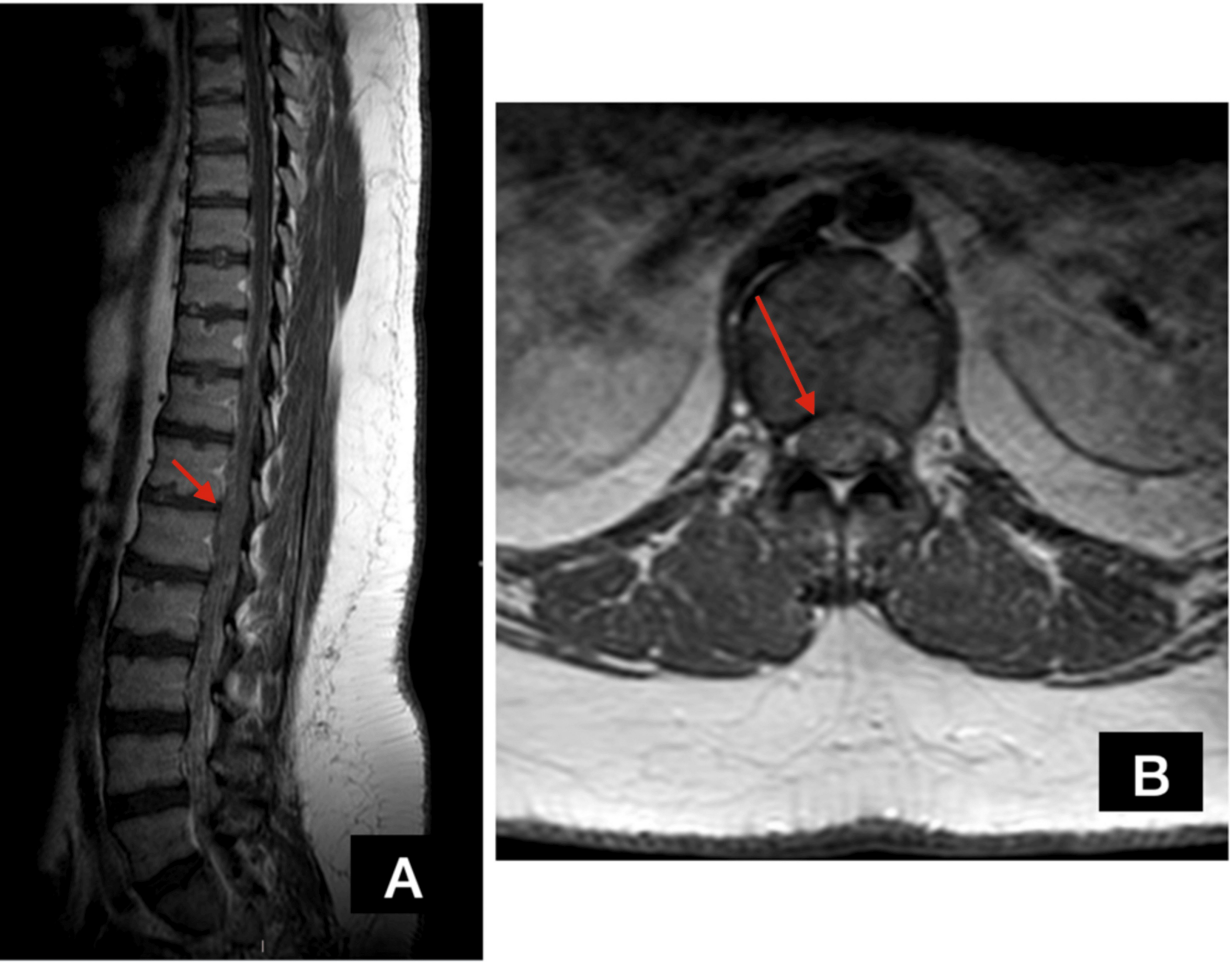
Post-op MRI scan findings. A: sagittal post-op, post-contrast T1 MRI scan; B: axial post-op post-contrast T1 MRI. Both scans demonstrate the complete removal of the lesion.

The patient was immediately transferred to the operating room, where, through T12 and L1 laminectomies, a myelotomy with subsequent pus removal was performed. The patient was started on broad-spectrum antibiotics (ceftazidime and clindamycin) until pending results of microbiological examination revealed Staphylococcus epidermidis and Corynebacterium spp. The infectious disease department modified his antimicrobial therapy accordingly, adding ciprofloxacin and sulfamethoxazole/trimethoprim. He started gradually regaining his muscle strength post-operatively (2/5 in all muscle groups in both lower limbs) while the hypoesthesia resolved completely. The post-op MRI demonstrated complete removal of the lesion (Figure 3), and the patient was discharged to a rehabilitation center in order to receive extended sessions of physiotherapy. On follow-up one year after surgery, he was ambulatory with slight signs of spasticity and no muscle weakness (MRC muscle power 5/5 in all muscle groups in both lower limbs), thus demonstrating total functional improvement.

## Discussion

Intramedullary abscess of the spinal cord (IASC) remains a rare entity, with less than 300 cases reported worldwide [3]. Nevertheless, it is a condition that should not elude the differential diagnosis of acute onset neurologic deficit mimicking spinal cord compression symptoms, since, as showcased in our report, with timely treatment we can offer our patients an excellent outcome. It should be highlighted that even after severe neurologic symptoms have developed, surgical evacuation and rehabilitation can dramatically improve the neurological function of our patients [3]. All patients with clinical suspicion of IASC should undergo an urgent MRI scan, as this examination remains the gold standard in diagnosing this condition [3] and should receive immediate broad-spectrum antimicrobial therapy [4,5]. Our case highlights the role of timely surgery, which not only offers a definite diagnosis but also mediates a complete and faster clinical improvement. However, since there are no guidelines regarding the role of surgery in IASC, antimicrobial therapy alone remains a viable option in patients with rapid neurological improvement or a lesion limited in size [2,3]. Recent literature suggests that surgery should always be considered when radiographic findings and clinical symptoms are consistent with IASC. Not only can surgery relieve symptoms of spinal cord compression, but it also provides an opportunity to obtain cultures that can guide antimicrobial therapy [3]. Unfortunately, there is no clear consensus on which treatment offers the best outcomes due to the rarity of this entity. Certain predisposing factors leading to the formulation of such abscesses have been recognized like diabetes, drug abuse, and immunosuppression but in our case, none of these were present [6]. A review of the literature suggests that an initial site of infection, often through hematogenous or contiguous spread, is typically associated with the formation of IASC. In this case, the patient had no other predisposing factors, and no alternative source of inflammation was identified. Given the identified source of inflammation, as confirmed by our initial CT scan showing diffuse opacification of the left mastoid cells, we hypothesize that this infection is related to the IASC. To our knowledge, this is the first reported case of IASC caused by hematogenous spread following otitis in a healthy adult.

## Conclusions

Intramedullary abscess of the spinal cord (IASC) remains a rare but treatable entity, with the role of surgery being critical to the outcome we can offer to our patients. Because of the rarity of such cases, a combined effort to report and categorize them should be made by the neurosurgical community. 
